# Discoidin Domain Receptor 1 Expression in Colon Cancer: Roles and Prognosis Impact

**DOI:** 10.3390/cancers14040928

**Published:** 2022-02-13

**Authors:** Kaouther Ben Arfi, Christophe Schneider, Amar Bennasroune, Nicole Bouland, Aurore Wolak-Thierry, Guillaume Collin, Cuong Cao Le, Kevin Toussaint, Cathy Hachet, Véronique Lehrter, Stéphane Dedieu, Olivier Bouché, Hamid Morjani, Camille Boulagnon-Rombi, Aline Appert-Collin

**Affiliations:** 1 Laboratoire de Biopathologie, Centre Hospitalier Universitaire de Reims, 51090 Reims, France; kaouther.ben.arfi@gmail.com (K.B.A.); camille.boulagnon@gmail.com (C.B.-R.); 2 UMR 7369, Matrice Extracellulaire et Dynamique Cellulaire (MEDyC), Université de Reims Champagne Ardenne (URCA), 51097 Reims, France; christophe.schneider@univ-reims.fr (C.S.); amar.bennasroune@univ-reims.fr (A.B.); nicole.bouland@univ-reims.fr (N.B.); lecuongbi@gmail.com (C.C.L.); kevin.toussaint@univ-reims.fr (K.T.); cathy.hachet@univ-reims.fr (C.H.); stephane.dedieu@univ-reims.fr (S.D.); 3 Laboratoire d’Anatomie Pathologique, Faculté de Médecine, 51100 Reims, France; 4 Unité d’Aide Méthodologique, Centre Hospitalier Universitaire, 51100 Reims, France; awolak-thierry@chu-reims.fr; 5 Unité BioSpecT, EA7506, Université de Reims Champagne Ardenne (URCA), 51096 Reims, France; guillaume.collin@univ-reims.fr (G.C.); veronique.lehrter@univ-reims.fr (V.L.); obouche@chu-reims.fr (O.B.); hamid.morjani@univ-reims.fr (H.M.); 6 Service d’Hépato-Gastroentérologie, Centre Hospitalier Universitaire, 51100 Reims, France

**Keywords:** colon cancer, discoidin domain receptor, prognosis, event free survival, survival

## Abstract

**Simple Summary:**

Colorectal cancer (CRC) is the third leading cause of cancer death in both sexes. Identification of the influencing factors and molecular mechanisms in CRC progression could improve patient survival. This study aimed first to characterize the expression of Discoidin Domain Receptor 1 (DDR1), a receptor tyrosine kinase for collagens in a large cohort of CRC patients, and second to establish in vitro whether DDR1 expression level is linked to CRC aggressiveness potential. Our immunohistochemical study indicated that DDR1 is highly expressed in colon cancer compared to normal colonic mucosa and its expression is associated with shorter event-free survival. In vitro, the invasive properties of several CRC cell lines seem to be correlated with the expression level of DDR1. Taken altogether, our results show that DDR1 is highly expressed in most colon adenocarcinomas and appears as an indicator of worse event free survival.

**Abstract:**

Extracellular matrix components such as collagens are deposited within the tumor microenvironment at primary and metastatic sites and are recognized to be critical during tumor progression and metastasis development. This study aimed to evaluate the clinical and prognostic impact of Discoidin Domain Receptor 1 (DDR1) expression in colon cancers and its association with a particular molecular and/or morphological profile and to evaluate its potential role as a prognosis biomarker. Immunohistochemical expression of DDR1 was evaluated on 292 colonic adenocarcinomas. DDR1 was highly expressed in 240 (82.2%) adenocarcinomas. High DDR1 immunostaining score was significantly associated, on univariate analysis, with male sex, left tumor location, *BRAF* wild type status, *KRAS* mutated status, and Annexin A10 negativity. High DDR1 immunohistochemical expression was associated with shorter event free survival only. Laser capture microdissection analyses revealed that DDR1 mRNA expression was mainly attributable to adenocarcinoma compared to stromal cells. The impact of DDR1 expression on cell invasion was then evaluated by modified Boyden chamber assay using cell types with distinct mutational profiles. The invasion capacity of colon adenocarcinoma is supported by DDR1 expression. Thus, our results showed that DDR1 was highly expressed in most colon adenocarcinomas and appears as an indicator of worse event free survival.

## 1. Introduction

Colorectal cancer (CRC) is ranked among the most common cancers in the world and is a significant public health issue in developed countries. Recent data indicated that CRC is the third most common cancer and the second leading cause of cancer death in both sexes [1]. The important mortality in CRC patients is highly correlated to its potential of metastasis reported in 50% of patients after surgery [2]. Indeed, about 39% of CRC patients are diagnosed at early stage with localized-stage disease. For these patients, the 5-year survival rate is 90%, while for the patients diagnosed with stage IV CRC, the survival declines to 12% [3].

However, at the same stage, all CRC do not have the same prognosis. Some parameters set by the tumor stage could refine the prognosis prediction and some histoprognosis factors have been identified: lymphovascular invasion, perineural invasion, tumor differentiation, or molecular profiles [2]. Treatment decisions could be influenced by these factors. In fact, many studies have been recently conducted to find new molecularly based prognostic markers, which are complementary to the data obtained by pathological diagnosis and therefore may increase the patient’s survival. However, new biomarkers able to stratify the prognosis groups of patients and improve treatment strategies remain necessary. For this purpose, several studies investigate the signaling pathways that promote the metastatic process in CRC in order to identify new key players in this process that could constitute potential targets [4].

Receptor tyrosine kinases (RTKs) play an important role in several cellular processes in tumors including growth, migration, invasion, and the response to therapies [5]. For instance, the mitogen-activated protein kinase (MAPK) pathway and the phosphoinositide-3-kinase–protein kinase B/Akt (PI3K-PKB/Akt) pathway, two main intracellular pathways activated by the epidermal growth factor receptor (EGFR), were the most used therapeutic targets in metastatic colon cancer [6].

Discoidin domain receptors (DDRs) are collagen receptors with tyrosine kinase activity. The expression of the two members of this family, DDR1 and DDR2, is different: DDR1 is preferentially located in epithelial cells whereas DDR2 is expressed more importantly in connective tissues of the embryonic mesoderm [7]. Both DDR1 and DDR2 are activated by fibrillar collagens such as type I collagen [8]. Several studies have suggested a pivotal role of DDRs in tumor progression [9,10,11]. DDR1 expression appears to be increased in a variety of tumors and is correlated to poor prognosis [9,10,11]. Indeed, high level of DDR1 expression has been observed in several tumors such as prostate [12], lungs [13], breast [14], and ovary [15], suggesting a potential role of DDR1 in tumorigenesis and tumor progression. Moreover, experimental models have demonstrated that DDR1 plays an important role in cell proliferation and the metastasis process [16,17,18,19].

However, its role appeared to be tumor dependent. DDR1 overexpression was associated with advanced tumor stages in esophageal cancer [20], brain tumors [21] and with poor survival, in lung adenocarcinoma [22] and serous ovarian cancer [15].

In colon carcinoma, the role of DDR1 remains incompletely elucidated. The prognosis impact of DDR1 in CRC had not been studied much until now. High DDR1 expression seemed to be associated with poor overall survival [23,24,25]. Moreover, Sirvent and co-workers have shown that DDR1 plays a key role in the invasion potential of CRC [26]. The pharmacological inhibition of DDR1-BCR signaling axis using nilotinib has indeed been reported to decrease invasion and metastatic processes in CRC. These results suggest that DDR1 could represent a potential target in CRC treatment [26].

In the present study, we evaluated the expression of DDR1 in a cohort of CRC that, to our knowledge, is the largest set of CRC specimens studied for this receptor up to date. Specifically, we assessed the association between DDR1 expression and associated clinicopathological and molecular characteristics and its potential value as a prognosis marker. Finally, we examined in vitro the role of DDR1 in cell invasion in several CRC cell lines to establish whether DDR1 expression level is linked to CRC aggressiveness potential.

## 2. Materials and Methods

### 2.1. Culture Cells

HCT116, SW480, SW620 and HT-29 colorectal carcinoma cell lines were purchased from American Type Culture Collection (ATCC, Rockville, MD, USA). HT-29DDR1-GFP and HT-29GFP were obtained as previously described [27]. All cell lines were grown in Dulbecco’s Modified Eagle Medium (DMEM) with high glucose (4.5 g/L) (Thermo Fisher Scientific, Villebon sur Yvette, France) containing 10% (*v*/*v*) fetal bovine serum (FBS) (Dutscher, Bernolsheim, France) and 1% penicillin-streptomycin (*v*/*v*, Invitrogen). Cells were regularly controlled for the absence of mycoplasma by PCR methods.

### 2.2. RNA Isolation from Cell Culture

Total RNA from cells was extracted as described previously [28] and single-stranded cDNA was synthesized from 250 ng total mRNAs using VERSO cDNA kit (Thermo Fisher Scientific, Waltham, MA, USA) according to the manufacturer’s instructions. Determination of the mRNA of DDR1 was carried out by real-time PCR as described [27].

### 2.3. Total Protein Extraction and Immunoblotting

Seventy-two hours after seeding, cells were washed with ice-cold phosphate-buffered saline (PBS) and harvested in lysis buffer (10 mM Tris, 150 mM NaCl, 5 mM EDTA, 1% triton, protease inhibitors (Roche Diagnostics, Indianapolis, IN), and 5 mM Na orthovanadate). Cell lysates were then centrifugated at 14,000 *g* for 10 min at 4 °C. Protein concentration was quantified by BCA Protein Assay Kit (Thermo Fisher Scientific, Waltham, MA, USA). Proteins were separated on acrylamide gels and electroblotted onto nitrocellulose membranes (Amersham Biosciences, Little Chalfont, UK). The blots were incubated with primary antibodies (DDR1 (D1G6), GFP (D5.1) and GAPDH (14C10)) and corresponding peroxidase conjugated secondary antibody as previously indicated [27].

### 2.4. Invasion Assay

Cell invasion was evaluated using type-I collagen-coated 24-well cell culture inserts with an 8 μm pore size (Dustscher, Bernolsheim, France). The Boyden chambers were coated with 25 μg cm^−2^ type-I collagen and then washed twice with PBS. A total of 5 × 104 cells were seeded into the upper chambers in a 200 μL DMEM culture medium, supplemented with 2% FBS, 1% penicillin–streptomycin. DMEM culture medium with 10% FBS and 1% penicillin–streptomycin was added in the lower chamber. After 24h, the chambers were washed with PBS, fixed with methanol and stained with Di Aminido Phenyl lndol (DAPI, Santa Cruz Biotechnology). Cells remaining on the upper face of the membranes were suppressed by scraping, and those on the lower side were counted after being imaged on the EVOS^®^ FL Auto Imaging System using a 40× objective (Thermofisher scientific, Waltham, MA, USA). Experiments were reproduced three times in triplicates. 

Concerning experiments using Nilotinib and DDR1-IN-1 inhibitors, 7.5 × 10^4^ cells were seeded into the upper chambers in a 200 μL DMEM culture medium, supplemented with 2% FBS, 1% penicillin–streptomycin in the presence or not of Nilotinib (100 nM, No.S1033) or DDR1-IN-1 (10 µM, No.S7498, Selleckchem). DMEM culture medium with 10% FBS and 1% penicillin–streptomycin was added in the lower chamber. After 24 h, the chambers were washed with PBS, fixed with methanol, and stained with crystal violet. Cells remaining on the upper face of the membranes were suppressed by scraping. Upon solubilization in acetic acid (10%), the amount of dye on the filter was quantified by spectrophotometry at 560 nm.

### 2.5. Patients

Patients and selection were clarified in paper from Boulagnon-Rombi et al. [29]. 

The study was conducted on adult patients who underwent surgery for sporadic colon cancer in the Digestive Surgery Department of the University Hospital of Reims between September 2006 and December 2012. Patients with rectal cancer were excluded.

Clinical data including age at the time of surgery, sex, performance status, surgical circumstances (tumor perforation, occlusion), tumor location, synchronous or metachronous metastases, tumor recurrence, treatment, death and pathological and molecular data including adenocarcinoma type, grade, and pTNM stage were collected. Patients were classified as having a right colonic cancer if the primary tumor was located in the caecum, ascending colon, hepatic flexure or transverse colon, and left colonic cancer if the tumor site was within the splenic flexure, descending colon, sigmoid colon, or rectosigmoid junction.

### 2.6. Pathology 

All colon adenocarcinomas were classified and subtyped according to The World Health Organization criteria [30] and staged according to the International Union Against Cancer 2009 guidelines [31]. Tumor budding was assessed on Hematoxylin- Eosin-Saffron slides and classified as low budding rate if less than 5 buds were present in the 0.785 mm^2^ hot spot [32].

### 2.7. Immunohistochemistry

Tissue samples were analyzed via tissue microarrays (TMA). For each tumor, 3 cores were punched in the central part and 3 cores at the invasive front of the tumor from the same original formalin-fixed paraffin-embedded tumor block. The cores were 2 mm in diameter and were precisely arrayed into a recipient paraffin block using the MiniCore Tissue Arrayer (Excilone, Elancourt, France). Sections of 4-μm thickness were cut and mounted on SuperFrost Plus Gold adhesive slides (Thermofisher Scientific, Waltham, MA, USA). Immunohistochemistry (IHC) was performed using DDR1 (D1G6) XP^®^ Rabbit mAb, rabbit Monoclonal antibody (1/100, Cell Signaling ref: #5583) after heat-induced epitope retrieval in citrate pH 6 buffer (95 °C, 40 min) and overnight antibody incubation at 4 °C and then visualized using 3-Amino-9-Ethylcarbazole (AEC). 

### 2.8. Scoring

Immunostaining intensity (SI) was graded independently by two pathologists (CBR, KBBA).

**Figure 1:**
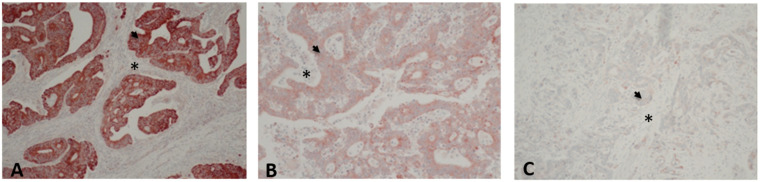
Representative images of DDR1 immunolabeling in colon adenocarcinoma. A. Strong and diffuse staining (red) in adenocarcinoma cells (arrow), (magnification ×10), scored 3+/high; B. Moderate and diffuse staining (red) in adenocarcinoma cells (arrow), (magnification ×20), scored 2+/high; C. Faint staining (red) in adenocarcinoma cells (arrow), (magnification ×10), scored 1+/low. Stromal cell highlighted by an asterisk (*) was weak (**A**) or negatively (**B**,**C**) stained in all cases.

Immunopositivity was defined as a brown cytoplasmic color in the tumor cells. Staining intensity was scored as follows: 0, negative staining signal in >50% of tumor cells; 1+, weak staining signal detected in >50% of tumor cells; 2+, moderate staining signal in >50% of tumor cells; 3+, strong staining signal in >50% of tumor cells (Figure 1). The staining intensity was then divided into score 0/1+ for low DDR1 expression or score 2+/3+ for high DDR1 expression as previously described [23].

### 2.9. Molecular Analyses

Tumor DNA was extracted and the mutation profile (*BRAF, KRAS,* and MSI status) of the samples was determined as described earlier [33].

### 2.10. Laser Capture Microdissection

Laser capture microdissection was performed on fresh frozen colon cancer specimens cut into 12 μm serial sections and mounted on PALM membrane slides (Zeiss, Oberkochen, Germany) as previously noticed [29].

RNA from tumor and stromal microdissected tissues were isolated and purified as indicated [29].

### 2.11. DDR1 mRNA Expression

Analysis of mRNA expression was performed as previously described [29]. Only RNAs with RQI values ≥5 were used for further analyses. Determination of the mRNA of DDR1 was carried out by real-time PCR as described [27].

### 2.12. Data Mining and Bioinformatic Analyses

Survival analyses were performed using publicly available data from TCGA, Martineau and SieberSmith gene expression dataset in the R2 microarray analysis and visualization platform (http://r2.amc.nl; last access date: 5 November 2021). The scan online algorithm was used to determine the cut-off values for separating high and low DDR1 expression groups.

### 2.13. Statistical and Survival Analyses

Statistical analyses and factors associated with immunohistochemical expression of DDR1 were clarified in paper from Boulagnon-Rombi et al. [29].

## 3. Results

### 3.1. Association of DDR1 Immunohistochemical Expression with Clinico-Pathological Features

The relationship between DDR1 expression and disease aggressiveness was investigated in a cohort of 292 colon cancer patients. The clinicopathological features are summarized in Table 1. The population consisted of 166 (57%) men and 126 (43%) women, whose mean age was 70.8 ± 10.8 years. Tumors were right-sided in 123 cases (42%), left-sided in 164 cases (56%), and multifocal in 5 cases (2%). The mean follow-up time was 43 months (±32 months).

Figure 1 illustrates representative IHC patterns of DDR1 expression. The immunostaining showed the localization of DDR1 mostly in the cytoplasm. The immunostaining intensity was strong in 144 (49.3%) samples, moderate in 96 (33%), and weak in 52 (17.8%), and no samples were found negative for DDR1 staining (score 0). DDR1 immunostaining was diffuse (>50% of positive tumor cells) in all cases. DDR1 immunolabeling in tumor stroma was weak or negative in all cases. For the statistical analysis, patients were divided into two groups: low expression of DDR1 for patients with immunostaining intensity scored 1 and high DDR1 expression for patients with immunostaining intensity scored 2 or 3. Thus, DDR1 expression by IHC was rated high in 240 (82.2%) cases. In case of samples presenting heterogeneity in immunostaining, the highest intensity was considered for scoring.

The relationship between DDR1 immunohistochemical expression and different clinicopathological and molecular characteristics was analyzed. Data are detailed in Table 2. In univariate analysis, a high DDR1 immunostaining score was significantly associated with male sex (*p* = 0.0195), left tumor location (*p* = 0.0114), BRAF wild-type status (*p* < 0.0001), KRAS mutated status (*p* = 0.0041), and absence of expression of the serrated markers Annexin A10 (*p* = 0.0097). In multivariate analysis, high DDR1 immunostaining score was independently associated with BRAF wild-type status only (*p* < 0.0001).

### 3.2. Survival Analysis

We next investigated the relation between DDR1 expression and prognosis. Univariate analysis demonstrated that age, tumor stage, vascular invasion, and metastasis were predictors of overall survival (OS) in our cohort (Table 3).

**Figure 2:**
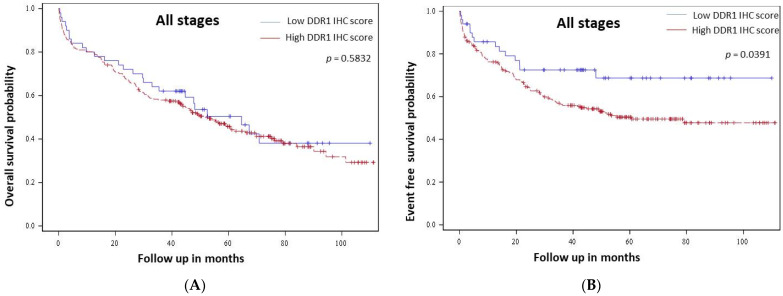
DDR1 value as a prognosis indicator in colon cancer patients from our cohort. Kaplan-Meier curves of overall survival (**A**) and event free-survival (**B**) probability for low (blue line) and high (red line) DDR1 immunohistochemical expression in adenocarcinoma cells from all tumor stages.

**Figure 3:**
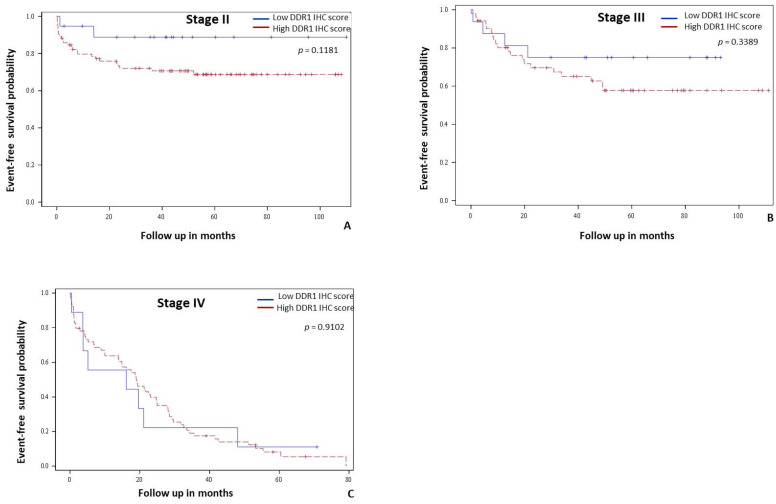
Stage specific event free survival analysis in colon cancer patients from our cohort according to DDR1 immunohistochemical expression. Kaplan-Meier curves of event free-survival probability for low (blue line) and high (red line) DDR1 immunohistochemical expression in cells in stage II (**A**), stage III (**B**) and stage IV patients (**C**).

High DDR1 immunostaining was not correlated with overall survival in all stages (*p* = 0.5832, Figure 2A) nor in metastatic (stage IV) patients (*p* = 0.8376, data not shown). Regarding event-free survival (EFS), univariate analysis revealed that occlusion, stage, vascular invasion, lymphatic invasion, differentiation grade, RAS status, CIMP status, and the level of DDR1 immunostaining scores were associated with shorter EFS (Table 3). High DDR1 expression was a predictor of shorter EFS in the entire cohort (*p* = 0.0391, Figure 2B). Stage specific analyses showed that DDR1 was not a predictor of EFS in stage II (*p* = 0.1181, Figure 3A), stage III (*p* = 0.3389, Figure 3B) and in metastatic patients (*p* = 0.9102, Figure 3C).

In our cohort DDR1 mRNA expression levels successfully evaluated in 66 patients were not correlated with OS (*p* = 0.86) nor EFS (*p* = 0.46), whatever the CCR stage (data not shown). 

**Figure 4:**
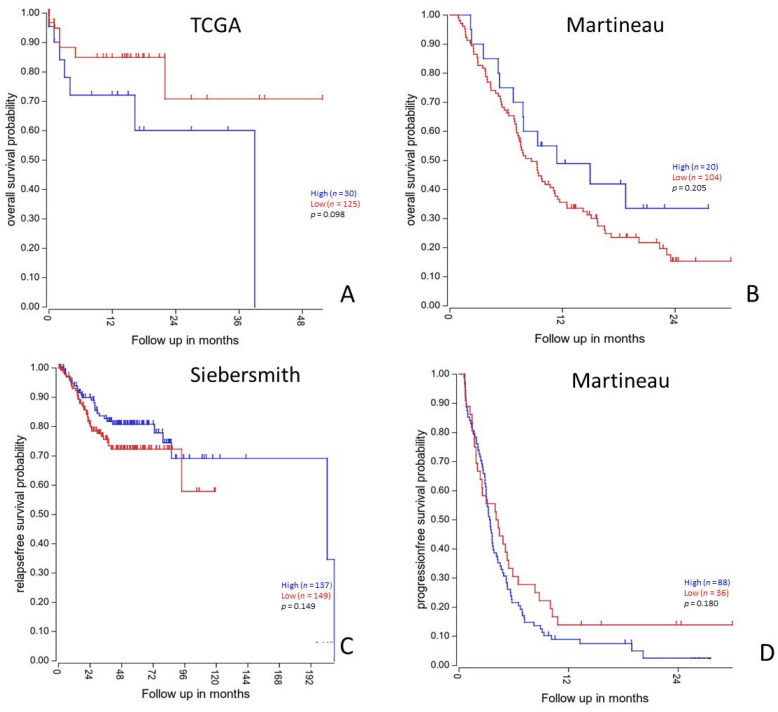
Survival analysis according to DDR1 mRNA expression profile in independent colorectal cancers patients’ cohorts. Kaplan-Meier curves of overall survival (**A**,**B**) and relapse or progression free-survival (**C**,**D**) probability for low (red line) and high (blue line) DDR1 mRNA expression in all stages colorectal cancers patients and in stage IV (metastatic) patients (**C**,**D**). Survival analysis and Kaplan Meyier curves of the TCGA, Martineau and SieberSmith gene expression dataset were obtained from R2 platform (http://r2.amc.nl; last access date: 5 November 2021). All *p*-values were calculated using R2 online tools.

To corroborate our previous results, we next performed survival analyses in SieberSmith (*n* = 286), Martineau (*n* = 124) [34] and TCGA cohorts (*n* = 174) obtained from R2 database [35,36]. In these cohorts, DDR1 mRNA expression was not correlated with overall nor relapse free survival (Figure 4).

### 3.3. DDR1 Is More Expressed in Tumor Cells Compared with Stromal Cells

**Figure 5:**
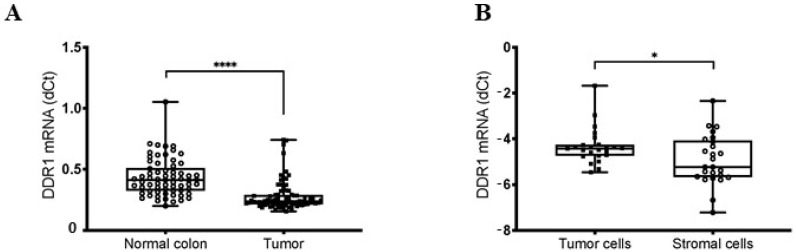
Comparison of DDR1 mRNA expression between tumor cells, normal colon and stromal cells. (**A**) Real-time PCR analysis of the DDR1 mRNA expression performed in colon adenocarcinoma and in normal colon mucosa fresh frozen samples. Values are represented as dCt normalized with RPL32. (**B**) Real-time PCR analysis of the DDR1 mRNA expression performed in adenocarcinoma cells and in stromal cells after laser capture microdissection. Values are represented as dCt normalized with RPL32. * *p* < 0.05, **** *p* < 0.0001, Mann Whitney test.

DDR1 mRNA expression has been determined by RT-qPCR on 65 colonic adenocarcinoma samples and 78 colonic mucosa samples. Surprisingly, data showed a significant decrease in DDR1 expression within tumor samples when compared with normal colon samples (Figure 5A). Due to the difference observed in DDR1 expression between stromal and malignant cells when evaluated by IHC analysis, we used Laser Capture Microdissection (LCM) to thereafter quantify DDR1 mRNA expression in tumoral and stromal areas of each sample as previously described [29]. LCM was performed on 25 colon adenocarcinoma samples and RT-qPCR revealed that DDR1 mRNA expression was higher in the tumoral area than in the stroma (Figure 5B).

### 3.4. DDR1 Mediates the Invasion of CRC Cells

**Figure 6:**
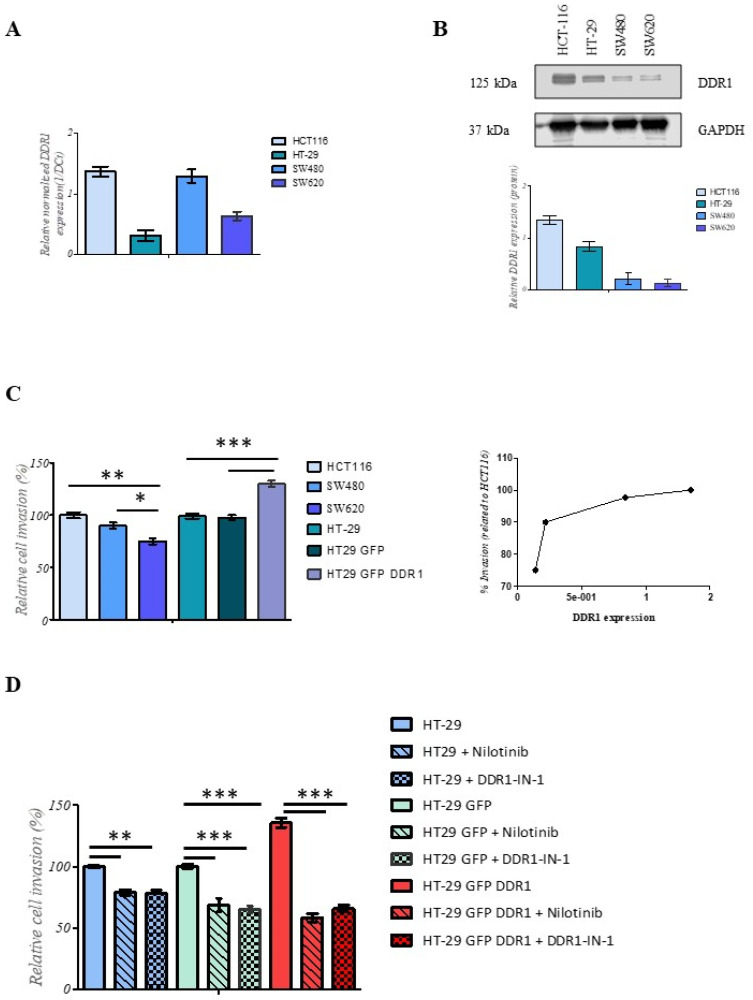
Human CRC cell invasion is modulated by DDR1 expression. (**A**) The relative mRNA expression of DDR1 was assessed using RT-qPCR. Values in HCT-116, HT-29, SW480, and SW620 were normalized with both RPL32 and RS18 mRNA expression. (**B**) The expression of DDR1 and GAPDH was assessed by western blotting using anti-DDR1 and anti-GAPDH antibodies in HCT-116, HT-29, SW480, and SW620 cells. Quantitative analysis of DDR1 protein was obtained by densitometry: the amount of DDR1 was normalized to GAPDH expression level (bottom panel). (**C**) HCT-116, HT-29, SW480, and SW620 were seeded into the collagen type I coated chambers for 24 h. Cells were then fixed with methanol and stained with DAPI. Results are expressed as mean ± SD of three independent experiments. Statistical significance was analyzed by a one-way ANOVA test using Dunnett’s multiple comparisons. * *p* = 0.05, ** *p* = 0.01, *** *p* = 0.001 as compared to HCT-116 cells or HT-29 cells. Correlation between DDR1 expression and cell invasion (right panel). (**D**) HT-29, HT-29^DDR1-GFP^, and HT-29^GFP^ cells were seeded into the collagen type I coated chambers for 24 h in absence or presence of nilotinib (100 nM) or DDR1-IN-1 (10 µM). Cells were then fixed with methanol and stained with crystal violet. Results are expressed as mean ± SD of three independent experiments. Statistical significance was analyzed by a one-way ANOVA test using Dunnett’s multiple comparisons. ** *p* = 0.01, *** *p* = 0.001 as compared to HT-29, HT-29^DDR1-GFP^, or HT-29^GFP^ cells.

We then investigated the possible role of DDR1 in CRC aggressiveness in vitro. We used HCT116, HT-29, SW480, and SW620 cell lines, which express different levels of DDR1 expression, and analyzed their ability to invade type I collagen as one of the main extracellular matrix components. These cell lines harbor different *KRAS*/*BRAF* statuses and their main characteristics are summarized in Supplementary Figure S2. The level of DDR1 expression was analyzed by both RT-qPCR and immunoblotting (Figure 6A,B, uncropped western blot images in Supplementary Figure S1). Data showed that the expression of DDR1 at the mRNA and protein levels was higher in HCT116 cells than in the other cell lines. In order to investigate deeply the impact of DDR1 on invasive properties of CRC cells, we used HT-29 cells expressing DDR1 at a basal level (HT-29^GFP^) and overexpressing the receptor (HT-29^DDR1-GFP^). As shown in Supplementary Figure S2, HT-29^DDR1-GFP^ expressed a high level of DDR1 when compared to wild-type HT-29 or HT-29^GFP^ cells. The invasion potential of CRC cell lines was evaluated based on modified Boyden chamber assay using type I collagen coating. Data showed that HCT116 cells exhibited a higher invasion rate than SW480 and SW620 cells. When DDR1 was overexpressed in HT29 cells (HT-29^DDR1-GFP^), the invasion rate was significantly increased compared to the control (HT-29^GFP^) (Figure 6C). Interestingly, the invasion rate positively correlated with DDR1 expression level. To confirm the role of DDR1 in the invasion process of colorectal cells, nilotinib (100 nM) and DDR1-IN-1 (10 µM) have been used to inhibit specifically DDR1. As shown in Figure 6D, significant inhibition of cell invasiveness was observed when the cells were treated with nilotinib or DDR1-IN-1 compared with the control ones. Overall, these data suggest that DDR1 is involved in CRC invasion phenotype and could be associated in this way with the worse event free survival.

## 4. Discussion

Many cancers are characterized by dysregulated expression of one or more RTKs. Such alteration has functional consequences at the cellular level which directly impact tumor progression, especially cell invasion and metastasis. DDRs play a key role in tumor progression, in part by regulating the reciprocal interplay between cancer cells and stromal collagens [37]. One of their major roles in the literature is their involvement in tumor invasion and metastasis [38].

In this study, we investigated the expression of DDR1 using immunohistochemistry in colon adenocarcinoma and studied the link between DDR1 expression with clinicopathologic and molecular parameters, including overall and event-free survival. Because DDR1 seems to play a role in CRC cell invasion and metastasis [5], we also investigated the impact of DDR1 on invasion properties of CRC cell lines in vitro using type I collagen as a main extracellular matrix component.

In this work, we showed that DDR1 expression was higher in adenocarcinoma cells than in normal colonic epithelium. DDR1 was highly expressed in a large majority (82.2%) of colon cancers. These results corroborate previous data showing a high DDR1 overexpression in 94% of colon cancer samples [23] and in tumor tissues from patients with primary CRC and hepatic CRC metastasis [24].

Our results demonstrated in univariate analysis that the clinico-pathological and molecular characteristics associated with DDR1 expression in colon adenocarcinoma were: male sex, left colon tumor localization, *BRAF* wild-type status, and absence of the expression of the serrated marker Annexin A10. 

To our knowledge, no study had investigated these clinico-pathological and molecular characteristics in association with DDR1 expression in colon adenocarcinoma, especially the potential association with the serrated pathway highlighted by its markers Annexin A10. The molecular profile associated with DDR1 high expression could be integrated into the CMS4 molecular subtype of colorectal cancer. These tumors are characterized by strong stromal infiltration and show clear upregulation of genes playing a role in epithelial mesenchymal transition and associated to transforming growth factor β (TGF β) signaling pathway, angiogenesis, matrix remodeling pathways, and the complement-mediated inflammation. These CMS4 tumors presented worse overall survival and relapse-free survival [39]. Indeed, DDR1 mRNA expression was not associated with any CMS subtype [25]. Our bioinformatic analyses revealed that high DDR1 mRNA expression was independently associated with worse OS and PFS in stage IV patients. Moreover, any significant association between DDR1 mRNA expression and OS or EFS has been found in our cohort of patients undergoing surgery for colonic adenocarcinoma. However, divergent results showed that DDR1 high mRNA expression was associated with worse OS whatever the tumor stage [24].

The major limitations of our study were its retrospective and single-center design and that few patients had DDR1 mRNA expression data. However, our results were validated with bioinformatic analyses in three other patients’ cohorts. In our patients’ cohort, DDR1 immunohistochemical expression was only associated with worse EFS whatever the stage. DDR1 high protein expression was not associated with OS or with stage specific EFS. In a previous immunohistochemical study, high DDR1 immunoreactivity score was correlated with a shorter overall survival in a cohort of 100 patients with colorectal cancer [23]. In this study, EFS was not evaluated and stage specific survival analyses were not performed.

The molecular mechanisms underlying the roles of the DDRs in various steps of colon carcinoma progression are largely undefined. To fill this gap, we investigated the potential role of DDR1 in tumor cell invasion by using several colorectal cancer cell lines that differentially express DDR1. In addition, HT-29 cells overexpressing DDR1 were established and led to enhanced cell invasiveness. The data showed that the tumor cell invasion capacity is closely correlated to DDR1 expression. Moreover, specific pharmacological inhibition of DDR1 with nilotinib and DDR-IN-1 significantly reduced HT-29 cell invasion. These results ascertained previous reports indicating DDR1 pro-invasive role in several tumor cell lines and DDR1 metastatic function in many cancers [12,17,19,40], and demonstrate the importance of DDR1 in invasive tumors. For instance, DDR1 expression is increased by the microRNA MiR-199a-5p and promotes invasion in CRC by activating epithelial-mesenchymal transition [41]. In human A375 melanoma, HT29 colon carcinoma and SK-HEP hepatoma cells, chemical inhibition or silencing of DDR1 reduces cell adhesion to collagen I and MMP-dependent invasion [42]. Recently, Romayor and coworkers have demonstrated that DDR1 expressed by tumor cells promotes hepatic cell ability to alter the ECM structure by regulating collagen and MMPs expression, thus suggesting an impact of DDR1 in the desmoplastic response of hepatic tumor microenvironment during CRC tumorigenesis [24]. 

In addition, it has been recently demonstrated that DDR1 can have a great impact on the invasion function of metastatic colon carcinoma [26]. Indeed, invasion and metastatic processes were decreased by DDR1-BCR signaling axis inhibition in vivo in colon carcinoma suggesting that DDR1 could be an effective therapeutic target in this cancer. The authors concluded that the inhibition of DDR1 kinase activity with nilotinib may be a therapeutic benefit in patients with advanced CRC [26].

In other cancers, DDR1 expression could also have a prognostic implication. Indeed, high expression of DDR1 has also been identified in 52.2% of hepatocellular carcinoma samples [43], 61.0% of non-small cell lung cancer [13], and 69% of serous ovarian cancer tissues [15]. Moreover, high DDR1 expression was more frequently expressed in invasive carcinoma than in bronchioloalveolar carcinoma in lung cancers and was associated with shorter overall survival in non-small cell lung carcinomas [22]. On the contrary, DDR1 was not associated with survival in prostate cancer [12] and low DDR1 expression was associated with triple negative subtype of breast cancer and with shorter survival in this cancer type [44].

Thus, the overexpression of DDR1 in these malignant diseases, particularly in colorectal cancer, supports the hypothesis that DDR1 upregulation is widespread in cancer and can play an important role in tumorigenesis and/or tumor invasion and metastasis.

## 5. Conclusions

In summary, DDR1 is highly expressed in colon cancer compared to normal colonic mucosa. This overexpression of DDR1 is found in a large majority of colon cancers, suggesting a role of DDR1 in colorectal carcinogenesis. Although DDR1 was associated with shorter EFS, its role as a prognosis marker remains uncertain. However, frequent high expression of DDR1 in colon cancer could be further explored as a potential therapeutic target in this indication.

## Figures and Tables

**Table 1:** Clinicopathological features of the cohort.

Clinicopathological Features	Total (%) *n* = 292
**Gender**	
Male	166(57)
Female	126 (43)
**Age (Mean ± standard deviation) years**	70.8 ± 10.8
**UICC stage**	
Stage I	34 (11.8)
Stage II	109 (37.8)
Stage III	72 (24.9)
Stage IV	74 (25.6)
**Tumor location**	
Left colon	164 (56)
Right colon	123 (42)
Multifocal	5 (2)
**Occlusion**	
Yes	34 (12)
No	258 (88)
**Tumor perforation**	
Yes	17 (6)
No	275 (94)
**Differentiation grade**	
Grade 1–2	245 (84)
Grade 3	47 (16)
**Annexin A10**	
Positive	36 (12)
Negative	255 (88)
***KRAS* status**	
Wild type	95 (67)
Mutant	46 (33)
***BRAF* status**	
Wild type	246 (86)
Mutant	40 (14)
**Microsatellite status**	
MSS	250 (87)
MSI	37 (13)
**CIMP status**	
No CIMP	20 (35.7)
CIMP-Low	30 (53.5)
CIMP-High	6 (10.7)

**Table 2:** Relationship between DDR1 expression and clinical and molecular characteristics.

Patients and Tumors Characteristics	*n*	DDR1		Univariate Analysis	Multivariate Analysis	
		High	Low	*p*-Value	OR [IC 95%]	*p*-Value
		*n* (%)	*n* (%)			
**Age (Years)**		70.23 ± 10.6	73.59 ± 11.2	0.052 *		
**Gender**				**0.0195 ^‡^**		n.s
Female	126	96 (40)	30 (57.7)			
Male	166	144 (60)	22 (42.3)			
**Tumor location**				**0.0114 ^‡^**		n.s
Left colon	164	143 (59.6)	21 (40.4)			
Right colon	128	97 (40.4)	31 (59.6)			
**UICC stage**				0.3240 ^‡^		
I	34	30 (12.5)	4 (8)			
II	109	90 (37.7)	19 (38)			
III	72	55 (23)	17 (34)			
IV	74	64 (26.8)	10 (20)			
**Differentiation grade**				0.0540 ^‡^		
1–2	245	206 (85.8)	39 (75)			
3	47	34 (14.2)	13 (25)			
**Vascular invasion**				0.2694 ^‡^		
Yes	115	90 (38.3)	15 (30)			
No	180	145 (61.7)	35 (70)			
**Perineural invasion**				0.6 ^‡^		
Yes	71	60 (25.5)	11 (22)			
No	214	175 (74.5)	39 (78)			
**Budding score**				1 ^†^		
High	14	12 (5.4)	2 (4.2)			
Low	254	208 (94.5)	46 (95.8)			
**CDX2**				0.0565 ^†^		
Positive	268	223 (94.9)	45 (86.5)			
Negative	19	12 (5.1)	7 (13.5)			
***KRAS* status**				**0.0041 ^‡^**		n.s
Wild type	95	69 (61.6)	26 (89.7)			
Mutant	46	43 (38.4)	3 (10.3)			
***BRAF* status**				**<0.0001 ^‡^**	**7.5 [4.11–13.67]**	**<0.0001**
Wild type	246	212 (90.2)	34 (66.7)			
Mutant	40	23 (9.8)	17 (33.3)			
**Microsatellite status**				0.0909 ^†^		
MSS	250	210 (89)	40 (78.4)			
MSI	36	25 (10.6)	11 (21.6)			
**CIMP status**				0.5488 ^†^		
No CIMP	20	18 (39.1)	2 (20)			
CIMP-L	30	23 (50)	7 (70)			
CIMP-H	6	5 (10.88)	1 (10)			
**Annexine A10**				**0.0097 ^‡^**		n.s
Negative	255	215 (90)	40 (76.9)			
Positive	36	24 (10)	12 (23.1)			

n.s: not significant; ^‡^: khi-2; ^†^: Fisher test; *: Satterthwaite.

**Table 3:** Analysis of factors associated with overall and event-free survival.

Variables	*n*	Overall Survival	Event Free Survival
		*p*-value	*p*-value
Age	281	**0.0046**	0.3824
Perforation (yes vs. no)	281	0.0003	**<0.0001**
Occlusion (yes vs. no)	281	<0.0001	**<0.0001**
T4 (T4 vs. T1, T2, T3)	281	**<0.0001**	**<0.0001**
N (0, 1a vs. 1b and N2)	281	**<0.0001**	**<0.0001**
Vascular invasion (yes vs. no)	274	**0.0002**	**<0.0001**
Lymphatic invasion (yes vs. no)	273	0.0622	**0.0264**
Stage UICC	278	<0.0001	**<0.0001**
Differentiation grade (yes vs. no)	283	0.0032	**0.00018**
CDX2 IHC expression (yes vs. no)	276	0.0245	0.8486
Metastasis (M0 vs. M+)	276	**<0.0001**	**<0.0001**
*KRAS* mutation (yes vs. no)	135	0.0689	**0.0010**
*BRAF* mutation (yes vs.no)	276	0.7616	0.2882
CIMP status (low vs. High)	53	0.0644	**0.0003**
Microsatellite status (MSS vs. MSI)	72	0.4009	0.2294
DDR1 IHC tumor score (low vs. High)	281	0.5832	**0.0391**

## Data Availability

The data presented in this study are available on request from the corresponding author.

## Supplementary Materials

The following are available online at https://www.mdpi.com/article/10.3390/cancers14040928/s1, Figure S1: (A) Summary table of the CRC cell lines characteristics. (B) DDR1 expression in HT-29, HT-29DDR1-GFP and HT-29GFP cell lines; Figure S2: Original, uncropped western blot scans.
