# Supplementary material for: Discoidin Domain Receptor 1 Expression in Colon Cancer: Roles and Prognosis Impact
Source: Cancers (Basel). 2022 Feb 13;14(4):928. doi: 10.3390/cancers14040928 (PMC8869771; doi:10.3390/cancers14040928)
Supplement: Supplementary file 1 [file cancers-14-00928-s001.zip › cancers-1516387-SI.pdf]

Figure S1

Supplementary Figure 6B

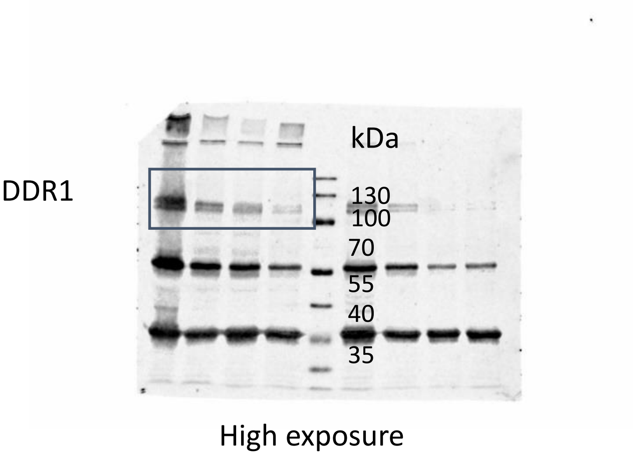

High exposure

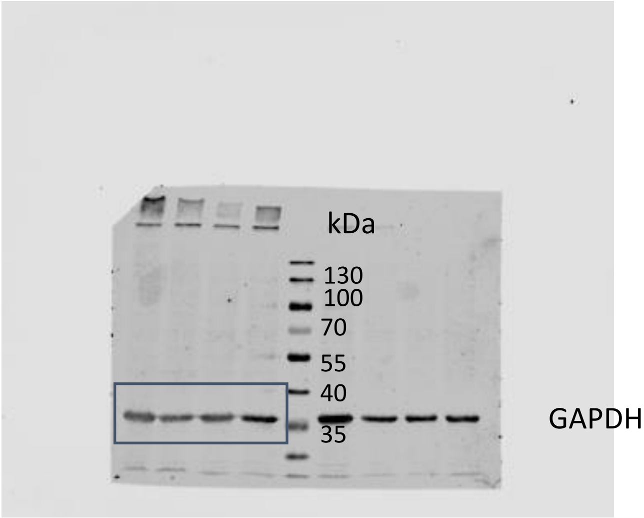

Low exposure

Densitometry readings/intensity ratio of Western blot bands found in Figure 6B

	DDR1	GAPDH	Rapport DDR1/GAPDH
**HCT-116**	34226	26050	1,313857965
**HT-29**	12874	16716	0,770160325
**SW480**	8192	25706	0,318680464
**SW620**	3756	30868	0,121679409

Supplementary Figure S2 Panel B

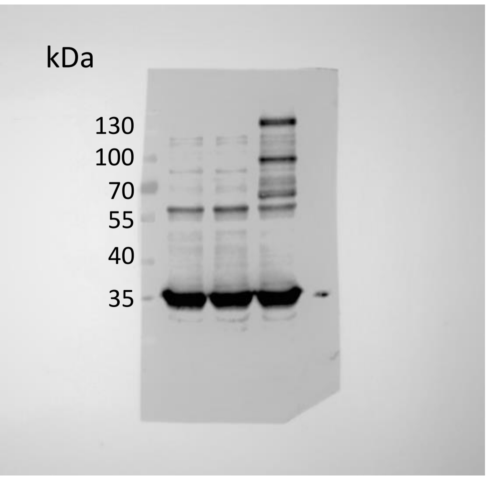

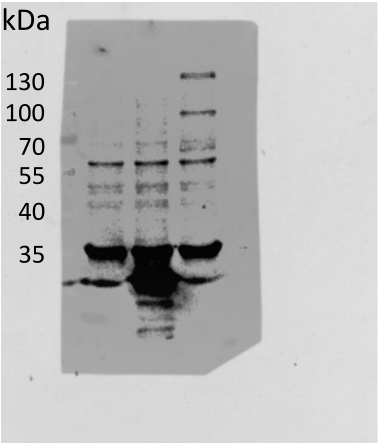

Figure S2

A

	HCT116	HT-29	SW480	SW620
**Patient**	48-Year-old male	44-Year-old female	50-Year-old male	51-Year-old male
**Disease**	Primary carcinoma	Primary adenocarcinoma	Primary adenocarcinoma	Metastatic adenocarcinoma (Lymph node)
**Stage**	Dukes'D	Dukes'C	Dukes'B	Dukes'C
BRAF	wt	V600E	wt	wt
KRAS	G13D	wt	G12V	G12V
P53	wt	R273H	R273H, P309S	R273H, P309S

B

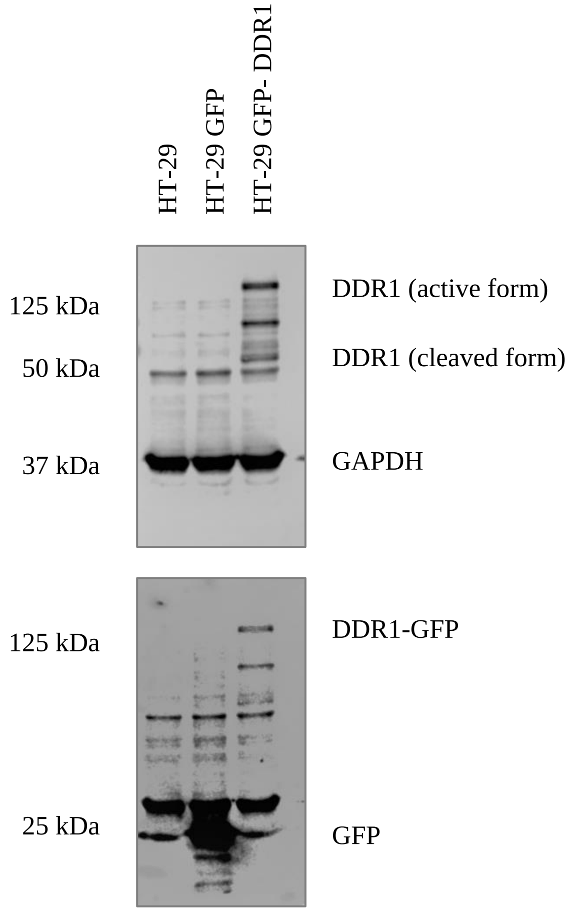

Figure S2. (A) Summary table of the CRC cell lines characteristics. (B) The expression of DDR1, GFP and GAPDH were analyzed by SDS PAGE followed by western blotting using anti-DDR1, anti-GFP and anti-GAPDH antibodies in HT-29, HT-29$^{DDR1\text{-}GFP}$ and HT-29^{GFP}
